# Comparative in vitro and in vivo evaluation of three tablet formulations of amiodarone in healthy subjects

**Published:** 2010

**Authors:** J. Emami

**Affiliations:** Department of pharmaceutics, School of pharmacy and pharmaceutical sciences, Isfahan Pharmaceutical Sciences Research Center, Isfahan University of Medical Sciences, Isfahan, Iran

**Keywords:** Bioequivalence, Bioavalability, Dissolution, Correlation

## Abstract

**Background and the purpose of the study:**

The relative in vivo bioavailability and in vitro dissolution studies of three chemically equivalent amiodarone generic products in healthy volunteers was evaluated in three separate occasions. The possibility of a correlation between in vitro and in vivo performances of these tablet formulations was also evaluated.

**Methods:**

The bioequivalence studies were conducted based on a single dose, two-sequence, cross over randomized design. The bioavailability was compared using AUC_0–72_, AUC_0–8_, C_max_ and T_max_. Similarity factor, dissolution efficiency (DE), and mean dissolution time (MDT) was used to compare the dissolution profiles. Polynomial linear correlation models were tested using either MDT *vs* mean residence time (MRT) or fraction of the drug dissolved (FRD) *vs* fraction of the drug absorbed (FRA).

**Results:**

Significant differences were found in the dissolution performances of the tested formulations and therefore they were included in the development of the correlation. The 90% confidence intervals of the log-transformed AUC0-72, AUC_0–8_, and C_max_ of each two formulations in each bioequivalence studies were within the acceptable range of 80–125%. Differences were not observed between the untransformed T_max_ values. Poor correlation was found between MRT and MDT of the products. A point-to-point correlation which is essential for a reliable correlation was not obtained between pooled FRD and FRA. The dissolution condition which was used for amiodarone tablets failed for formulations which were bioequivalent in vivo and significant difference between the dissolution characteristics of products (*f*2<50) did not reflect their in vivo properties.

**Major conclusions:**

Bioequivalence studies should be considered as the only acceptable way to ensure the interchangeability and in vivo equivalence of amiodarone generic drug products. The dissolution conditions used of the present study could be used for routine and in-process quality control of amiodarone tablet formulations.

## INTRODUCTION

Amiodarone, [2-butyl-3-(3,5-diiodo-4-β-diethyl- aminoethoxybenzoyl) benzofuran], initially used as an antianginal (1), is now widely used in the management of severe ventricular and supraventricular arrhythmias (2). It belongs to the class III anti-arrhythmic drugs, which prolong the duration of the action potential and the effective refractory period in both atria and ventricles (2). Amiodarone is absorbed variably and erratically from the gastrointestinal tract. Oral bioavailability ranges from 22 to 86% (3, 4). The low solubility of amiodarone in aqueous solution and hepatic first pass effect which has not been clearly defined might be a reason for its unpredictable absorption (5, 6). Amiodarone belongs to class II biopharmaceutical classification system (BCS) which is characterized by high membrane permeability and slow dissolution rate due to low aqueous solubility(7).

Certain changes in the formulation, the manufacturing process, the site of manufacture and the scale-up of the manufacturing process may alter the dissolution rate and the bioavailability of drug from solid dosage forms. Therefore, bioavailability issues have been of increasing concern to drug regulatory authorities for assessment of the safety and efficacy of drug products. Local drug regulatory authorities have, therefore, issued guidelines to ensure adequate bioavailability studies in new drug applications for synonym drugs (8).

While the pharmacokinetics, metabolism, and bioavailability of amiodarone have been studied (2–6), the bioequivalence of the marketed products has not been described and no information about in vitro-in vivo correlation (IVIVC) of this drug is available.

One of the challenges of biopharmaceutics research is correlation of the in vitro drug release information of various drug formulations to the in vivo drug profiles (9). For amiodarone, a correlation between dissolution rate and the in vivo performance might be expected (8–10).

Since drug release pattern from each dosage form was analyzed on a µ-bondapack C (150×4.6 is significantly affected by the special design which is employed in manufacturing of the formulations, planning an in vitro dissolution method corresponding to the in vivo drug absorption rate, will facilitate the development of the drug formulations and quality control tests (9–11). For the development of an ideal oral formulation of amiodarone, it would be highly desirable to have an appropriate dissolution method which could predict the progress of the drug release and the in vivo release rate of the drug. An official monograph of amiodarone tablet formulation does not exist in any accredited pharmacopoeia and appropriate dissolution conditions have not yet been described for this drug. However, a dissolution method for amiodarone tablet is recently proposed by FDA. Therefore, an appropriate dissolution conditions based on in vivo performance could be more confidently adapted for routine and in-process quality control studies.

This paper describes the result of the bioequivalence study and dissolution behavior of six (three reference and three test tablet formulations) immediate release amiodarone tablet formulations. Since the in vitro dissolution characteristics of these tablets exhibited different release pattern, the possibility of correlation between in vitro dissolution data and in vivo bioavailability of these tablet formulations was also investigated.

## MATERIAL AND METHODS

### 

#### Materials

Products under the study were six amiodarone immediate-release tablet formulations containing 200 mg amiodarone hydrochloride which exhibited different release pattern due to variation in proprietary manufacturing procedure. For tests T1, T2, T3 and for References R1, R2, and R3 were designated for identification. Amiodarone hydrochloride was obtained from Sigma (St. Louis, MO, USA), the internal standard, trifluoperazine was from SmithKline Beecham Inc. (Philadelphia, PA, USA), hydrochloric acid, tribasic sodium phosphate, monobasic sodium phosphate, orthophosphoric acid 85%, sodium laurylsulphate, ammonium acetate, sodium acetate, acetonitrile, and hexane, were from Merck (Germany), methanol and acetonitrile were from Caledon (Canada, Ontario). All reagents and solutions of this study were analytical grade except methanol and acetonitrile which were HPLC grade.

#### Methods

##### Drug content uniformity in tablet formulations

From each formulation, 20 tablets were transferred into a 50-ml volumetric flask containing 25 ml of methanol, sonicated, diluted with methanol to volume, mixed and filtered. Samples were assayed by a HPLC method developed in this laboratory. Briefly, an aliquot of 25 µl of clear sample solution mm) column, using acetonitrile-methanol (1:1) and 0.05 M monobasic potassium phosphate solution in deionized water (90:10, final pH, 3.5) at 243 nm. Drug contents of samples were determined by calibration curve.

##### Dissolution studies

The release characteristics of tested formulations were determined using USP Apparatus II (Pharma Test, PTZWS3, Germany) at 75 rpm in 900 ml of acetate buffer (pH=5, 0.1 M) containing 1% SLS maintained at 37±0.2°C. Dissolution tests were performed on 12 tablets at time intervals of 0, 5, 10, 15, 30, 45, 60, 75, 90, 105, and 120 minutes using, 5 ml samples. Samples were filtered and analyzed spectrophotometrically for amiodarone concentration in order to characterize the dissolution profiles.

##### Bioavailability studies

Three separate bioequivalence studies (study 1, 2, and 3) on three commercial generic products (T1, T2, and T3) were conducted during 2000–2005. For each study, twelve healthy adult male volunteers were recruited. Age (years), weight (kg) and height (cm) of participants in the study 1 were 22–26, 59–79, and 168–192, for the study 2 were 20–27, 65–90, 165–184 and for the study 3, were 19–25, 62–87, 165–185, respectively. The studies were approved by the ethics committee on human studies of the Isfahan University of Medical Sciences. On the basis of medical history, clinical examinations and laboratory tests, no subject had a history and evidence of hepatic, renal, gastro-intestinal or hematological disorders or any acute or chronic disease or drug allergy. The subjects were instructed to abstain from taking any medication at least 2 weeks prior to and during the study period. Informed consent was obtained from the subjects after explaining the nature and purpose of the study. The protocol was the conventional, randomized, two-way cross- over bioequivalence study with twelve subjects in each treatment group. In the first trial period, after an overnight fasting, subjects were given a single dose of either formulation (reference or test) in a randomized fashion with 200 ml of water. Food and drinks (other than water, which was allowed after 2 hrs) were not allowed for 4 hrs after dosing to all volunteers. Approximately 10 ml of blood samples were drawn into tubes through an indwelling canola before (0 h) and at 1, 2, 3, 4, 5, 6, 7, 8, 12, 24, 36, 48, 60 and 72 hrs after dosing. The blood samples were centrifuged at 3000 rpm for 15 min and serum samples were separated and kept frozen at -200C in coded glass tubes until analysis.

##### HPLC assay

A reversed phase HPLC method was developed in home to quantitate serum levels of amiodarone. Chromatographic separation was performed using a µ-Bondapak C18 (250×3.9 mm, Waters, Ireland) column. The mobile phase consisted of acetonitrile- 0.01 M KH PO solution (70/30) containing 20 µl triethylamine with the final pH of 4.0. The aqueous phase was eluted at a flow rate of 1.5 ml/min and effluent was monitored at 243 nm. To 1 ml of either blank serum spiked with different amount of amiodarone (calibration samples) or serum of volunteers in a 10 ml test tube, were added 50 µl of internal standard solution, trifluoperazine, 1 ml of sodium acetate buffer (1 M, pH=5.4), and 8 ml of hexane and vortexed and centrifuged at 2000 g for 3 min. The supernatant was separated and evaporated to dryness under nitrogen gas. The residue was reconstituted with 100 µl of mobile phase and 50 µl aliquot was injected into the HPLC column. The standard curve covering 10–500 ng/ml concentration range was linear, the inter- and intra- day precision and accuracy were less than 10%, the limit of quantification was 10 ng/ml, and theextraction efficiency was between 92% to 95% for calibration standard concentrations.

##### Dissolution data analyses

The in vitro drug release profiles of each two dosage forms (test versus reference) were compared using the similarity factor, *f2*, as described in FDA guidance for dissolution testing (13). Dissolution efficiency (DE) was used for comparison of dissolution rates and calculated as explained previously (12).

Mean dissolution time (MDT) was considered as a basis for the dissolution rates and was used to establish the correlation with in vivo mean residence time (MRT).

MDT was estimated by moment analysis as applied previously to tablet formulations (14). Dissolution rate constants (K) were calculated, assuming first order kinetics for fast dissolution products (r2 ranging 0.942–0.986), from the slope of natural logarithm of the remaining percentage to be released versus time (15). The time at which the dissolution process was complete calculated from 0.693/K_d_

##### Pharmacokinetic analyses

The pharmacokinetic parameters were calculated by non-compartmental methods. The elimination rate constant (k_E_) was obtained from the least square fitted terminal log-linear portion of the plasma concentration-time profile. The area under the curve to the last measurable concentration (AUC_0-t_) was estimated by the linear trapezoidal rule. The area under the curve extrapolated to infinity (AUC_0–8_) was calculated by equation of AUC_0-t_+C_t_/k_E_ where C_t_ is the last measured concentration. The peak plasma concentration (C_max_) and corresponding time to peak (t_max_) were determined by inspection of the individual drug serum concentration-time profiles (8).

##### Statistical analyses

For the purpose of bioequivalence analysis, AUC_0-t_, AUC_0–8_, C _max_ and T_max_ were considered as primary variables. For each of parameters, by an analysis of variance (ANOVA) procedure for cross-over design the values obtained for the two products were determined to assess the effect of treatment (formulation), periods, sequences, and subjects on the parameters (16). A difference between two related parameters was considered statistically significant for a *P* value of less than 0.05. The 90% confidence intervals of the pharmacokinetic parameters of the two products were also estimated. The AUC and C_max_ values were logarithmically transformed prior to the analysis (17). Non-parametric Wilcoxon signed rank test for paired samples, was used to compare values of Tmax of the test over the reference products. The inter-subject variation of AUC_0-t_, AUC_0–8_, and C_max_ parameters was estimated by calculation of the respective coefficient of variation (CV) using the mean square error obtained from the ANOVA procedure. All statistical analyses were performed on untransformed data using SPSS 10.

##### Correlation development

The correlation was developed using the data of mean amiodarone serum concentration *vs* time following ingestion of six formulations. The principles of statistical moment analysis were utilized to assess the correlation. In this level of correlation, the in vitro MDT of the product was compared to in vivo MRT. MDT was calculated as pointed out earlier and MRT was calculated using equation described by Gibaldi and Perrier (18). An approach based on cumulative fraction absorbed was also utilized to achieve an IVIVC. Prior to development of the IVIVC, the fraction of the drug dissolved (FRD) was determined using the aforementioned dissolution testing methods. In addition, the fraction of absorbed amiodarone (FRA) of each formulation was estimated by the Wagner-Nelson method. The cumulative fraction of the absorbed drug absorbed at time t was calculated as follows:

1 $$F_{t}=\frac{C_{t}+K_{E}\int_{0}^{t}\mathrm{Cdt}}{K_{E}\int_{0}^{\infty }\mathrm{Cdt}}$$

where, C_t_ is serum concentration at time t and K_E_ is elimination rate constant (18).

Linear correlations between FRD and FRA were established for pooled mean data of formulations from following equation:

2 $$y=y_{0}+\mathrm{ax}$$

where, y_0_ and α represents the regression parameters, *y* is FRA, and *x* is FRD. For the model the F-test and r were determined.

## RESULTS AND DISCUSSIONS

### 

#### In vitro studies

All products met the general pharmaceutical specifications for weight variation, content assay and content uniformity assay.

**Figure 1:**
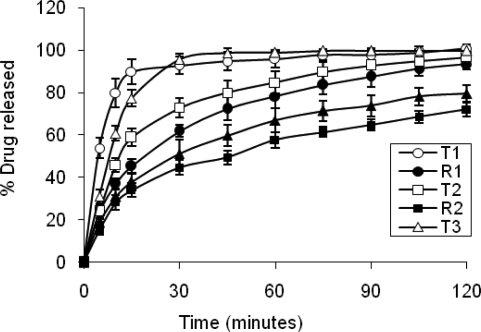
Profiles of mean dissolution rate of tablet formulations of amiodarone (references and tests) performed in acetate buffer (pH=5) containing 1% SLS at 75 rpm employing USP apparatus paddle method. Results are expressed as mean±SD (n =12) of% total amiodarone content.

Figure 1 shows the dissolution profiles of both reference and test tablet formulations which exhibited relatively immediate release behavior. The CV% associated with the dissolution data at each sampling time and for each formulation was less than 10%. In general, under the above described dissolution condition, test tablets had a higher dissolution rate than the reference formulations. This observation was supported by statistical analyses comparing the DE of test and reference tablets profiles at 45 min (p<0.05).ANOVA revealed significant differences in DE values for all tablet formulations (P<0.05) except for R1 versus R3 (DE, 49.6±2.4 vs 41.1±3.6; P=0.226). Products T1 and T3 showed faster dissolution rates reflected in greater DE (82.6±3.5 and 75.1±1.4, respectively); while T2, R2 and R3 exhibited slower dissolution rates as indicated in lower DE (58.8±2.5, 35.8±2.6, and 41.1±3.9, respectively). On the basis of dissolution efficiencies of the tested products, significant differences were found in their dissolution performances and therefore were included in the development of IVIVC.

#### In vivo studies

**Figure 2:**
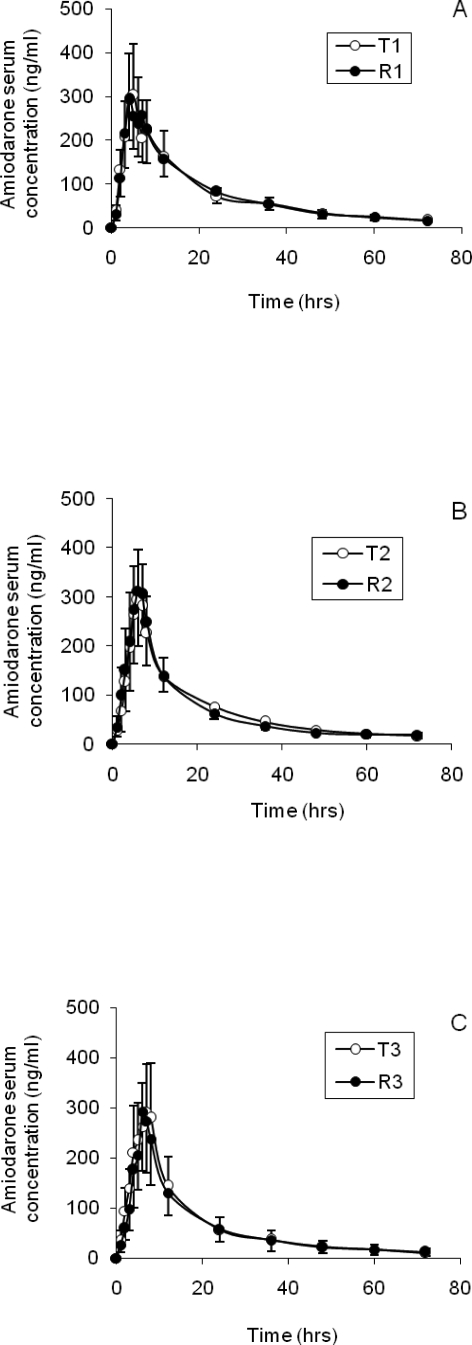
The mean serum amiodarone levels *vs* time profiles following ingestion of a single dose of the test and reference tablet products to 12 healthy volunteers. Data is shown as mean±SD. A: bioequivalence study 1, B: bioequivalence study 2, C:bioequivalence study 3.

The concentration-time profile following oral administration of each of the two amiodarone hydrochloride tablet preparations (test *vs* reference) are depicted in figure 2 A, B, and C. The mean pharmacokinetic parameters for the brands of amiodarone hydrochloride tablets are also summarized in table 1.

**Table 1:** Pharmacokinetic parameters of the three bioequivalence studies performed on each of the two products of amiodarone tablets (test and reference) administered orally to 12 healthy volunteers.

Prameters	T1 Mean±SD	R1 Mean±SD	CI_90%_	*P* value	Intra Subject CV%	T2 Mean±SD	R2 Mean±SD	CI_90%_	*P* value	Intra Subject CV%	T3 Mean±SD	R3 Mean±SD	CI_90%_	*P* value	Intra Subject CV%
**C _max_(µg/ml)**	329±128	350±112	0.909–1.041	0.548	9.8	342±92	354±85	0.963–1.020	0.753	5.2	311±93	342±118	0.931–1.018	0.344	6.5
**AUC_0–72_(µg.h/ml)**	5588±1854	5625±1973	0.947–1.058	0.931	8.1	4934±1433	4807±1245	0.981–1.046	0.391	5.7	4876±1603	4645±1836	0.982–1.070	0.324	6.7
**AUC_0–8_(µg.h/ml)**	5811±191	5833±2078	0.946–1.059	0.957	8.2	5291±1486	5100±1296	0.982–1.053	0.351	6.1	5043±1631	4865±1847	0.973–1.065	0.508	6.51
**T_max_(hrs)**	5.42±1.47	5.17±1.64	-	0.691	-	6.15±0.55	6.08±0.76	-	0.739	-	7.00±0.74	6.67±0.89	-	0.465	-

The AUC_0–72_, AUC_0–8_, C_max_, and T_max_, for each pair of products (test *vs* reference) in three separate bioequivalence studies were not statistically different (P>0.05), suggesting that the serum profiles generated by reference tablets were comparable to those produced by the test product (Table 1). Moreover, 90% confidence intervals of the AUC_0–72_, AUC_0–8_, C_max_ of the two formulations in each set of studies were found to be within the relative bioavailability acceptable range of 80–125% (Table 1). Wilcoxon signed rank test did not show any difference between the untransformed values of Tmax of the test compared to the reference products. The intra-subject CV for AUC_0–72_, AUC_0–8_, C_max_ appeared to be small. On the basis of the above analysis the test products could be considered bioequivalent with references.

#### In vitro - In vivo relationship

An appropriate dissolution conditions based on in vivo performance could be adapted for routine and in-process quality control of amiodarone tablet formulations. It was of interest, therefore, to explore if condition of dissolution of this study, which is very similar to what is proposed by FDA, correlates with serum plasma profiles already obtained by performing bioavailability studies.

**Figure 3:**
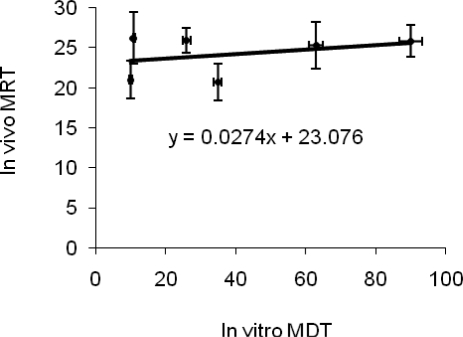
Correlation between the mean dissolution time (MDT) (calculated from in vitro dissolution data) and mean residence time (MRT) (calculated from serum drug concentration data). Data represent the mean of twelve determinations standard deviation.

Four correlation levels have been defined as Level A, B, C, and multiple-level C (9, 19). Statistical moment analysis has been suggested as a better parameter to examine IVIVC (9). In addition, a level B correlation uses all in vitro and in vivo data and was therefore employed between MRT and MDT. A poor correlation (p=0.033) between MRT and the in vitro MDT for the six products was found in the present study (Figure 3).

An approach based on FRA and FRD from the dosage forms was utilized to test a level A correlation. The last sampling time for in vitro dissolution was 120 minutes and the in vivo time points of up to 18 hrs were included in to correlation.

**Figure 4:**
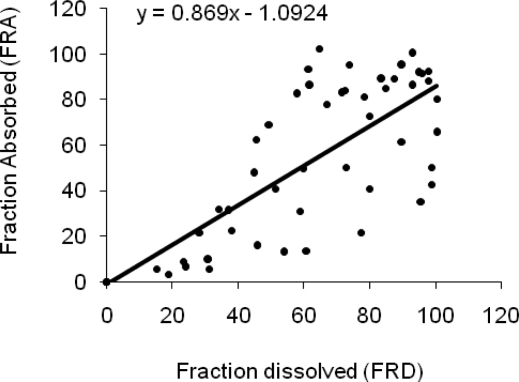
In vitro-in vivo correlation between the fraction dissolved in vitro (FRD) and the cumulative fraction absorbed in vivo (FRA) for the conventional amiodarone tablet formulations under study. Each point represents the mean of twelve determinations.

Finally, figure 4 was constructed by plotting FRD versus FRA pooled data of the six products to give the following equation.

y=0.928x-11.12

A point-to-point correlation which is the cornerstone of an acceptable and reliable correlation was not achieved. In addition, using the pair-wise procedure based on similarity factors (*f2*) which is a model independent approach, the *f2* values of (25.4%), (29.2%) and (25.5%) were calculated for studies, numbers 1, 2 and 3 respectively. Profiles were considered similar, where *f2* values lie between 50–100%. As *f2* values were not in the acceptance range, the products were considered not equivalent with respect to their in vitro release characteristics (Figure 1). Since dissolution of the drug from the test products were faster than that of the reference formulations, values of similarity factor were smaller. In order to find out a predictive in vitro dissolution method both similarity factor of in vitro dissolution testing and regression parameters of IVIVC should be taken into account. The dissolution condition which was used for amiodarone tablet formulations in the present study failed for formulations that are bioequivalent in vivo. Also very significant difference was observed between the dissolution characteristics of R1 and T1 (*f2=*25.4%, DE of 49.6 ±3.5 *vs* 82.6±3.5; P<0.05), R2 and T2 (*f2=*29.2%, DE of 35.7±2.6 *vs* 58.8±2.5; P<0.05) and R3 and T3 (*f2=*25.5%, DE of 41.1 ±3.9 vs 75.9±2.1; P<0.05). These products were bioequivalent in vivo as shown in Table 1. It seems that the dissolution medium of the present study does not completely simulate GI tract conditions. It is reported that biorelevant dissolution media could be used successfully to predict the in vivo performance of poorly water-soluble drugs. Strong correlations have been obtained with the poorly soluble drugs when of biorelevant dissolution medium is used (20). Although in most cases the in vivo differences amongst MRT of the various formulations were statistically significant, they were not considerably significant to yield a strong correlation between MRT and MDT. Further studies are suggested with conventional and more sustained- release formulations using biorelevant dissolution medium. At a dose of 200 mg amiodarone HCl and aqueous solubility of 0.5 mg/ml, 400 ml of fluid are required to dissolve one dose of amiodarone HCl which results in a dosing number (D_0_) of 1.6 (10). Therefore, the volume of water which is taken initially with the dosage form will dissolve the drug to a great extent and reduces the dependency of drug absorption to the drug dissolution. This may result in a poor correlation which was obtained in this study.

## CONCLUSION

From the results of the present study, it may be concluded that bioequivalence studies should be conducted as the only acceptable means to ensure the interchangeability and in vivo equivalence of amiodarone generic drug products. The dissolution condition of the present study could be used for routine and in-process quality control of amiodarone tablet formulations.
